# Prognostic value of gross tumor volume delineated by FDG-PET-CT based radiotherapy treatment planning in patients with locally advanced pancreatic cancer treated with chemoradiotherapy

**DOI:** 10.1186/1748-717X-7-37

**Published:** 2012-03-19

**Authors:** Cem Parlak, Erkan Topkan, Cem Onal, Mehmet Reyhan, Ugur Selek

**Affiliations:** 1 Baskent University Adana Medical Faculty, Department of Radiation Oncology, Kisla Saglik Yerleskesi, Adana, Turkey; 2 Baskent University Adana Medical Faculty, Department of Nuclear Medicine, Kisla Saglik Yerleskesi, Adana, Turkey; 3 American Hospital- University of Texas M.D. Anderson Radiation Oncology Center, Istanbul, Turkey

**Keywords:** Locally advanced pancreatic carcinoma, Chemoradiotherapy, FDG-PET-CT based RTP, Gross tumor volume

## Abstract

**Background:**

We aimed to assess whether gross tumor volume (GTV) determined by fusion of contrast-enhanced computerized tomography (CT) and 18F-fluoro-deoxy-D-glucose positron emission tomography-CT (FDG-PET-CT) based radiotherapy planning could predict outcomes, namely overall survival (OS), local-regional progression-free survival (LRPFS), and progression-free survival (PFS) in cases with locally advanced pancreas cancer (LAPC) treated with definitive concurrent chemoradiotherapy.

**Methods:**

A total of 30 patients with histological proof of LAPC underwent 50.4 Gy (1.8 Gy/28 fractions) of radiotherapy concurrent with continuously infused 5-FU followed by 4 to 6 courses of maintenance gemcitabine. Target volume delineations were performed on FDG-PET-CT-based RTP. Patients were stratified into 2 groups: GTV lesser (GTV_L_) versus greater (GTV_G_) than cut off value determined by receiver operating characteristic (ROC) analysis, and compared in terms of OS, LRPFS and PFS.

**Results:**

Median GTV delineated according to the FDG-PET-CT data was 100.0 cm^3^. Cut off GTV value determined from ROC curves was 91.1 cm^3^. At a median follow up of 11.2 months, median OS, LRPFS and PFS for the entire population were 10.3, 7.8 and 5.7 months, respectively. Median OS, LRPFS and PFS for GTV_L _and GTV_G _cohorts were 16.3 vs. 9.5 (*p *= 0.005), 11.0 vs. 6.0 (*p *= 0.013), and 9.0 vs. 4.8 months (*p *= 0.008), respectively.

**Conclusions:**

The superior OS, LRPFS and PFS observed in GTV_L _patients over GTV_G _ones suggests a potential for FDG-PET-CT-defined GTV size in predicting outcomes of LAPC patients treated with definitive C-CRT, which needs to be validated by further studies with larger cohorts.

## Background

Concurrent chemoradiotherapy (C-CRT) has been a well-established treatment option for patients with locally-advanced pancreas cancer (LAPC), which constitutes approximately one third of pancreatic carcinomas (PC) [1,2]. In spite of significant improvements in diagnostic imaging, chemotherapy, and radiotherapy (RT), outcome in LAPC is still dismal with median overall survival (OS) rarely exceeding 1 year even following aggressive C-CRT [3,4].

Several surgical series have established factors including tumor size, status of resection margins, invasion of vascular and/or adjacent structures, degree of differentiation, performance status, carbohydrate antigen 19-9 and C-reactive protein levels, to affect the outcome in resectable PC [2]. However, there are only few series indicating prognostic factors in the LAPC [4-7], while none -to the best of our knowledge- has specifically evaluated local/regional tumor burden in terms of gross tumor volume (GTV) in the era of definitive C-CRT, which was shown to be highly predictive for local control (LC), OS and progression-free survival (PFS) in various primaries including, lung, oral cavity and nasopharynx [8-11].

In studies by Lemke et al. [12] and Delbeke et al. [13] functional imaging with 18F-fluoro-deoxyglucose positron emission tomography (FDG-PET) has been demonstrated to increase accuracy of PC staging compared to conventional methods with its higher sensitivity and specificity. Based on these promising results, and considering the fact that accurate definition of primary tumor and its local/regional extensions is the first step of any effort to improve LC rates with RT, in our earlier comparative dosimetric study, we demonstrated an average of 29.7% enlargement in GTV being necessary in 5 out of 14 (35.7%) patients due to detection of additional computerized tomography-(CT) occult lymph node metastases and/or primary tumor extensions outlined by co-registered FDG-PET-CT [14]. From thereon, we changed our routine practice for target volume delineation that was based solely on contrast-enhanced CT findings, and began to use fusion of contrast-enhanced CT and FDG-PET-CT for this purpose in patients with LAPC.

In this study, which involved the first 30 patients of our on-going phase II study targeting to enrol a total of 120 patients, we investigated whether size of GTV determined by contrast-enhanced CT/FDG-PET-CT fusion could predict treatment outcomes, namely OS, local-regional progression-free survival (LRPFS), and PFS in LAPC patients treated with C-CRT.

## Methods

### Patients population

Thirty consecutive patients, referred with histologically proven diagnosis of surgically unresectable LAPC from March 2008 to December 2010 were enrolled in this study. Our institutional definition for technically unresectable PC is to be stage III (T4N0-1 M0) disease, which is the involvement of celiac axis and/or superior mesenteric artery. Disease extent was determined in all patients with radiological studies and laparotomy or laparoscopy. Standard radiological studies included contrast-enhanced abdominal CT, magnetic resonance imaging (MRI) and/or MR-cholangiopancreaticography (MRCP). All patients were re-staged via fusion of previous CT images (obtained ≤ 1 week before PET/CT scans) with FDG-PET-CT obtained for RTP. All patients underwent laparoscopic (n = 12) or laparotomic (n = 18) examination and biopsies for histologic diagnosis of primary tumor and enlarged/metabolically active regional lymph nodes and isolated single organ metastasis respecting the current standard institutional staging procedure for pancreatic carcinoma. Patients, who had received chemotherapy or abdominal irradiation previously, were not included in the study. The eligibility criteria also included an age of 18 to 70 years, Eastern Cooperative Oncology Group (ECOG) performance status (PS) of 0 to 2, presence of measurable or evaluable lesion, no contraindication for FDG-PET-CT imaging, an adequate bone marrow reserve (hemoglobin value of ≥ 10 g/dL, leucocyte of ≥ 4.000 μL, and thrombocyte of ≥ 100.000 μL), hepatic (aspartate aminotransferase or alanine aminotransferase of < 5 times the upper limit) and renal function (serum creatinine < 2 mg/dL). All patients signed informed consent, and the study design was approved by the Institutional Ethical Committee, in accordance with the Helsinki Declaration and Rules of Good Clinical Practice.

### FDG-PET-CT image registration and radiation treatment planning

FDG-PET-CT scans were performed according to the institutional protocol described elsewhere [14]. Areas of FDG uptake were categorized as malignant based on location, intensity, shape, size, and visual correlation with contrast-enhanced CT images to differentiate physiologic from pathologic uptake.

Image registration and RTP were performed via Eclipse 7.5 (Varian Medical Systems, Palo Alto, CA, USA) RTP system. The use of standard uptake values (SUV) alone for determining malignant involvement for delineation is subjective; therefore SUV measurements were not specifically used alone for delineation. For all 30 patients, two radiation oncologists consensually defined the target volumes, with the assistance of a nuclear medicine physician, and contoured the GTV, the planning target volume (PTV), and the organs at risk (OAR) on the contrast-enhanced CT/FDG-PET-CT fusion images. While the GTV (primary and nodal) was delineated on the FDG-PET/CT fusion, all OAR volumes were contoured from the CT due to inherent difficulty with edge detection in contouring PET volumes. For each patient, GTV included the primary tumor (GTV_P_) and involved lymph nodes (GTV_N_) apparent on contrast-enhanced CT (short axis ≥ 1.5 cm) and/or PET images. Those nodes < 1.5 cm were involved in GTV only if they were judged to be malignant on PET scan. Based on the literature [15] depicting nearly 1.5 cm movement of pancreas with respiration and considering the unavailability of motion tracking system and image guidance at our department, PTV was defined by adding 2 cm to GTV at all directions except for intersecting OAR restrictions to allow for microscopic extension, organ motion and set-up errors. Elective nodal irradiation was not permitted in the study.

### Treatment delivery

A single target volume with no cone down volumes was intended to be treated. A four-field technique (postero-anterior, antero-posterior, and laterals) was mandated, and treatment volumes were defined by using customized multi-leaf collimators. All patients received the RT protocol utilizing 18 MV photon energy linear-accelerators. A dose of 50.4 Gy (1.8 Gy/fr) was prescribed to encompass the defined PTV with isodose lines between 95% and 107%. To achieve this, we used dosimetric practice wedges to modify beams. Dose-volume histograms were generated for each patient to assess target volume coverage and organ at risk doses. The maximum dose limits for normal tissues were 45 Gy for spinal cord; 50 Gy for small bowel and stomach; 50 Gy for ≤ one-third, 35 Gy for two-thirds, and 30 Gy for three-thirds of the liver; and 20 Gy for at least two-thirds of one functioning kidney.

All patients received continuously infused 5-FU (225 mg/m^2^/day, 7 days/week) throughout the RT course (for 5.5 weeks), and additional 4 to 6 courses of maintenance gemcitabine (1,000 mg/m^2 ^IV over 100 min, days 1 and 8, every 21 days) following C-CRT.

### Toxicity assessment

Patients were assessed weekly or if necessary more frequently during C-CRT, every 3 months for the first 2 year, and every 6 months, thereafter. Early and late clinical toxicity was recorded and blood was collected for haematology and biochemistry assays at each assessment. Toxicity was assessed and scored with the aid of CTC 3.0 (Common Toxicity Criteria).

### Response evaluation and follow-up

Response to treatment was assessed by re-staging FDG-PET-CT scans, carried out 12 weeks after the completion of the treatment, according to EORTC-1999 guidelines [16]. Thereafter, patients were monitored by 8-12 weekly studies (blood count/chemistry; serum CEA and CA 19-9). Additional abdominal ultrasound and/or CT, chest CT, cranial magnetic resonance imaging, and FDG-PET-CT were used as indicated.

### Statistical analyses

The primary aim of this study was to evaluate predictive usefulness of GTV on clinical outcomes. For this purpose, we used receiver operating characteristic (ROC) analysis to determine whether GTV improved discrimination of outcomes. The ROC analysis represents the area under the curve (AUC) of sensitivity versus false-positive rate (1-specificity), and is equivalent to the probability that a predictive model will assign a higher probability of an event to subjects who subsequently have an event. Then, patients were dichotomized into two groups, GTV greater (GTV_G_) versus lesser (GTV_L_) than the cut off value, and compared in terms of LRPFS, PFS, and OS. LRPFS was defined as survival without local-regional failure, calculated as the time between the first day of treatment and the date of local-regional failure or death/last visit. PFS and OS were calculated as the time between the first day of treatment and any type of disease progression, and the date of death/last visit, respectively. Survival analysis was performed using the Kaplan-Meier method, and survival curves were compared with two-sided log-rank tests. *P *≤ 0.05 was considered statistically significant.

## Results

Pretreatment characteristics of all 30 patients enrolled to the study are as summarized in the Table 1. All cases received the prescribed dose of RT plus the scheduled chemotherapy concomitantly.

**Table 1:** Patient characteristics

Characteristic	Value
**Age (Years)**	
**Median**	57
**Range**	39-68
**Gender (%)**	
**Male**	21 (70)
**Female**	9 (30)
**Performance Status (%)**	
**ECOG 0-1**	23 (76.7)
**ECOG 2**	7 (23.3)
**Location of Tumor (%)**	
**Head**	23 (76.7)
**Body**	7 (23.3)
**Clinical Stage (%)**	
**T4N0**	13 (43.3)
**T4N1**	17 (56.7)
**SUVmax**	
**Median**	14.5
**Range**	6.2-22.6
**GTV (cm^3^)**	
**Median**	100
**Range**	32.3-224.3
**GTV_P _(cm^3^)**	
**Median**	93.4
**Range**	32.3-205.1
**GTV_N _(cm^3^)**	
**Median**	7.7
**Range**	0-19.2

**Abbreviations**: *ECOG *Eastern cooperative oncology group; *GTV *gross tumor volume; *GTV_N _*nodal gross tumor volume; *GTV_P _*primary gross tumor volume; *SUV *standard uptake value

Median GTV delineated by utilizing the contrast-enhanced CT/FDG-PET-CT fusion data was 100.0 cm^3 ^(range, 32.9 cm^3 ^to 224.3 cm^3^). From the ROC analysis, the best cut-off point was set at 91.1 cm^3 ^where sensitivity and specificity were 79.6% and of 91.7, respectively. The area under the ROC curve was 77.7. In 13 cases (43.3%), GTV was lower than 91.1 cm^3^.

**Figure 1:**
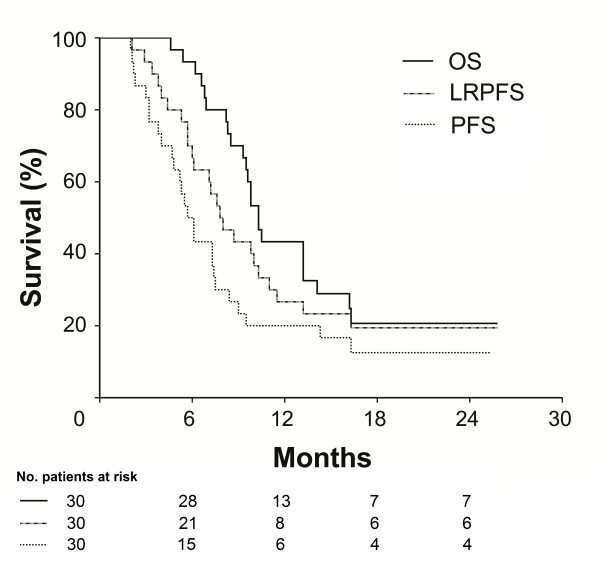
**Survival curves for whole study population**. Solid line: Overall survival (OS); Dashed line: Progression-free survival (PFS); Doted line: Local-regional progression-free survival (LRPFS).

At a median follow up of 11.2 months (range, 4.6 to 25.8 months), 23 out of 30 evaluable cases (76.7%) died. Six cases were alive with no disease progression, 4 of whom were from the GTV_L _group while remaining one was still alive with hepatic metastases. Median follow up was 14.1 months (range, 6.9 to 25.8 months) for GTV_L _and 9.5 months (range, 4.6 to 25.3 months) for GTV_G_. Median OS, LRPFS, and PFS for the entire population were 10.3 months (95% CI: 9.3 to 11.3 months), 7.8 months (95% CI: 5.8 to 9.8 months), and 5.7 months (95% CI: 4.6 to 6.7 months), respectively (Figure 1).

**Figure 2:**
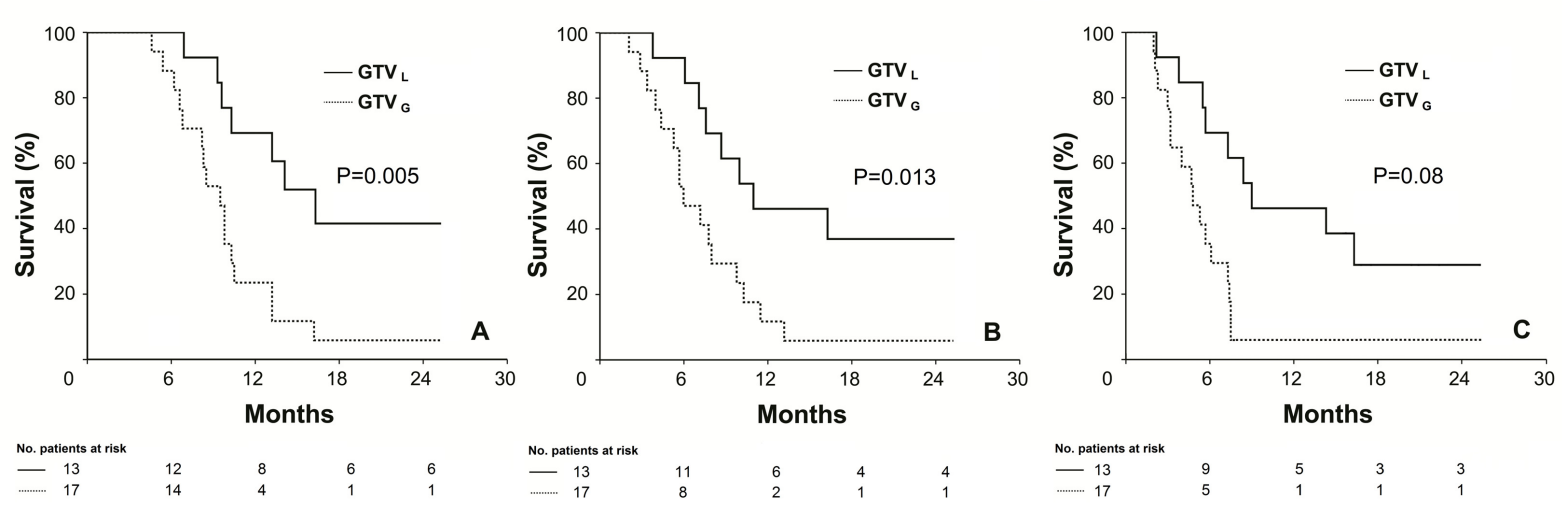
**Comparative survival analyses between GTV_L _and GTV_G _cohorts**. A: Overall Survival (OS); B: Local Regional Progression-free Survival (LRPFS), C: Progression-free Survival (PFS);. Solid line: GTV_L_; Dashed line: GTV_G_.

Comparisons of survival data by Log-rank test revealed that patients in group GTV_L _had significantly longer OS, LRPFS, and PFS than those in GTV_G _(Figure 2) (*p *= 0.005, 0.013 and 0.008, respectively). Corresponding median OS, LRPFS, and PFS for the cohorts GTV_L _versus GTV_G _were 16.3 [95% CI, 11.6 to 21.0] versus 9.5 (95% CI, 8.0 to 11.0), 11.0 (95% CI, 2.6 to 19.4) versus 6.0 months (95% CI, 4.1 to 7.9), and 9.0 (95% CI, 0.8 to 17.2) versus 4.8 (95% CI, 3.1 to 6.5), respectively. Results of univariate analyses for OS, LRPFS, and PFS were given in Table 2.

**Table 2:** Univariate analyses for survival

Characteristics	N	Median OS	P	Median LRPFS	P	Median PFS	P
		Months (95%CI)		Months (95%CI)		Months (95%CI)	
**GTV**							
**GTV_L_**	13	16.3 (11.6-21.0)	0.005	11.0 (2.6-19.4)	0.013	9.0 (0.8-17.2)	0.008
**GTV_G_**	17	9.5 (8.0-11.0)		6.0 (4.1-7.9)		4.8 (3.1-6.5)	
**GTV_N_**		-					
**< Median**	15	13.2 (8.2-18.2)	0.085	9.8 (7.3-12.3)	0.18	8.4 (4.9-11.9)	0.086
**≥ Median**	15	9.5 (7.511.5)		6.0 (3.3-8.7)		4.8 (3.2-6.4)	
**Nodal status**							
**N0**	13	13.2 (7.8-18.6)	0.25	9.8 (7.5-12.1)	0.36	8.4 (5.2-11.6)	0.25
**N1**	17	9.8 (7.8-11.8)		6.1 (4.1-8.1)		4.8 (3.1-6.5)	
**Age**							
**< Median**	14	10.5 (7.2-13.8)	0,99	7.6 (6.1-9.1)	0.97	5.7 (4.6-6.8)	0.92
**≥ Median**	16	9.8 (9.0-10.6)		7.8 (2.5-13.1)		5.7 (0.8-10.6)	
**Gender**							
**Male**	21	10.3 (9.0-11.6)	0.86	7.6 (6.3-8.9)	0.52	5.7 (4.5-6.9)	0.89
**Female**	9	10.3 (8.8-11.8)		10.3 (3.0-17.6)		7.3 (2.6-12.0)	
**ECOG**							
**0-1**	23	10.3 (9.2-11.4)	0.61	8.0 (5.7-10.3)	0.84	5.3 (3.1-7.5)	0.88
**2**	7	10.3 (7.7-12.9)		7.6 (6.3-8.9)		7.3 (3.2-11.4)	
**Location of Tumor**							
**Head**	23	10.3 (9.1-11.5)	0.58	7.6 (4.6-10.6)	0.52	5.7 (4.8-6.6)	0.31
**Body**	7	10.5 (8.7-12.3)		8.7 (6.4-11.0)		7.3 (2.2-12.4)	

**Abbreviations**: *ECOG *Eastern cooperative oncology group; *GTV *gross tumor volume; *GTV_G _*gross tumor volume greater than 91.1 (cm^3^); *GTV_L _*gross tumor volume lesser than 91.1 (cm^3^); *GTV_N _*nodal gross tumor volume; *LRPFS *local regional progression-free survival; *OS *overall survival; *PFS *progression-free survival

During follow-up 24 (80%) patients relapsed. Initial failure site was distant in 21 (70%) and local in 3 (10%) patients. The latter 3 patients also subsequently developed distant metastases during their follow up. Details of initial recurrence pattern according to the GTV groups are summarized in Table 3. Eventually, 16 (53.3%) patients developed infield recurrences with accompanying distant metastases. No isolated marginal or regional failure was reported as an initial or ultimate site of disease progression.

**Table 3:** Patterns of initial disease progression (N = 30)

Site	GTV_G _(N:17)	GTV_L _(N:13)	Overall (N:30)
	N (%)	N (%)	N (%)
**Local**	3 (17.6)	0 (0)	3 (10)
**Regional**	0 (0)	0 (0)	0(0)
**Distant**	13 (76.5)	8 (61.5)	21 (70)
**Liver**	3 (17.6)	3 (23.1)	6 (20.0)
**Peritoneum**	2 (11.8)	1 (7.6)	3 (10.0)
**Brain**	0 (0)	1 (7.6)	1 (3.3)
**Multiorgan**	8 (47.1)	3 (23.1)	11 (36.7)
**Total progression**	16 (94.1)	8 (61.5)	24 (80.0)

**Abbreviations**: *GTV_G _*gross tumor volume greater than 91.1 (cm^3^); *GTV_L _*gross tumor volume lesser than 91.1 (cm^3^)

Rates of acute toxicities experienced in the study are listed in Table 4. In general, all 30 patients were able to tolerate the C-CRT with no Grade 4/5 acute toxicity. Unplanned treatment breaks was mandated in 4 patients (13.3%) with an average of 3.8 days (range; 2 to 6 days), due to grade 3 toxicities; diarrhea in 2, leukopenia in 1, and vomiting in 1 patient. Because of Grade 3 diarrhea refractory to conventional loperamide and Grade 3 leukopenia, hospitalization was needed in 2 respective patients. However, all 4 patients were able to complete the planned C-CRT course after symptomatic and supportive treatment. At long-term, 3 patients (10.0%) developed symptoms of grade 3 gastric outlet obstruction at median 5.1 months (range; 3.1 to 8.7 months) after completion of C-CRT. Although late toxicity cannot be excluded as a cause, because of simultaneous evidence of disease progression in 2 patients at 4.4 and 7.1 months, progressive disease was probably associated with the symptoms in these two patients. Two additional (6.6%) patients experienced grade 2 gastric ulcer at 10.6 and 14.7 months, respectively, which were successfully managed with appropriate medication. No patients developed liver or renal dysfunction attributed to this C-CRT protocol.

**Table 4:** Frequency of Grade 3 acute toxicities

Toxicity	Grade		
	
	0-1	2	3
**Nausea**	24	4	2
**Vomiting**	26	3	1
**Diarrhea**	24	4	2
**Tumor pain**	28	2	0
**Anorexia**	20	10	0
**Fatigue**	26	3	1
**Gastritis**	28	2	0
**Leukopenia**	21	7	2
**Anemia**	23	6	1
**Thrombocytopenia**	24	5	1

## Discussion

We have investigated the predictive utility of GTV delineated by utilizing co-registered contrast-enhanced CT/FDG-PET-CT on outcomes in patients with LAPC treated with definitive C-CRT, and demonstrated that patients with smaller GTVs had significantly better OS, LRRFS and PFS compared to larger counterparts.

One of the reasons triggering our project is the impact of the size of the primary pancreatic tumors [17], which has been well-established in large surgical series of resectable PC demonstrating a better outcome with smaller sizes [18-20]. In their series, Birk et al. [18] and Sohn et al. [19] respectively demonstrated that patients with tumors < 2 and < 3 cm had significantly superior outcomes compared to those with 2-4 and ≥ 3 cm. In another study of 1697 patients from Johns Hopkins Hospital, de Jong et al. [20] also evaluated the impact of tumor size on survival following pancreaticoduodenectomy, and showed that 5-year OS was inversely proportional to tumor size (≤ 2 cm: 28.8% vs. 2-5 cm: 19.4% vs. ≥ 5 cm: 14.2%, p < 0.001), and that the size correlated with the risk of other adverse factors, with larger tumors being more likely to be associated with nodal disease and poor differentiation (*p *< 0.05).

Although tumor size at largest dimension has been addressed in a limited number of studies [21-24], to the best of our knowledge, the impact of three dimensional local/regional tumor burden expressed as GTV on outcome in the era of unresectable LAPC treated with definitive C-CRT has not been formally evaluated yet. In this respect, although two studies appeared to directly evaluate prognostic utility of the GTV size on outcome in LAPC patients [25,26], our study differs from them in significant aspects. In the first study, Rwigema et al. [25] successfully treated a group of LAPC patients including primary, recurrent, resected but margin positive and metastatic disease with stereotactic body radiotherapy (SBRT). This was a true volumetric study similar to one presented here, and found 15 mL as a significant volumetric cut point for survival difference. In the second study reported recently, Bjerregaard et al. [26] evaluated the impact of GTV on outcomes of initially unresectable 176 patients with LAPC. In this study, the authors chose to group patients by 25 cm^3 ^increments in GTV, and reported a survival advantage favoring the patients with smaller GTVs. However, although their results are in line with ours, there are remarkable differences between these two studies and the one presented here: First, Rwigema and colleagues used SBRT, which have significantly different radiobiological effects on tumor tissue than fractionated 3D conformal RT utilized here, and although our population size is relatively smaller, it is still more homogenous since we did not include resected, recurrent or metastatic patients. And second, Bjerregaard and colleagues studied on a highly heterogeneous study population, which included of 72 T3N0, 71 T3N1, and only 33 T4NX patients. Therefore, it is difficult to generalize their results for T4N0-1 patients as they constituted less than 20% of the study population. Likewise, authors chose to group patients by somehow arbitrarily specified 25 cm^3 ^increments in GTV rather than using possibly more relevant ROC defined cut off point(s), which creates further difficulties in interpretation of outcomes.

Herein, we preferred 3D tumor volume to define the tumor burden based on the studies suggesting that one-dimensional tumor measurements may not be as representative of tumor size as true volume calculations, especially in irregularly shaped tumors. In patients with advanced head and neck cancer, Rudat et al. [27] reported precision and reliability of the CT based volume measurements by repeated measurements using irregularly shaped phantoms. Furthermore, Titola et al. [28] proposed that one-dimensional volume estimation of irregularly shaped tumor-like phantoms should be substituted by true computer-based volume calculations.

The GTV, investigated here, has also been found to be of highly prognostic significance in various tumor sites. Bradley et al. [8] reported GTV to be a prognostic factor on both univariate and multivariate analysis in 207 NSCLC patients treated with definitive 3D conformal RT with or without chemotherapy, while Etiz et al. [9] pointed out the importance of total tumor volume (< or ≥ 80 cm^3^), defined by combination of primary tumor and nodal volume, on OS in irradiated inoperable NSCLC patients. Predictive role of GTV, delineated by 3D conformal RTP, on outcomes has also been demonstrated in nasopharyngeal [10], hypopharyngeal [29,30] and other head and neck sites [11,31,32]. Likewise, we have studied to pioneer the importance of well-defined GTV in the setting of LAPC, and our results revealed that median OS, LRRFS and PFS for the cohort with GTV lower than and greater than the ROC-defined 91.1 cm^3 ^cut value were 14.1 versus 9.5 months, 10.0 versus 6.0 months, and 8.4 versus 4.8 months, respectively (*p *< 0.05 for each).

Based on the assumption that the majority of the benefit from RT would result from control of the primary tumor, rather than subclinical disease in lymph nodes, which could potentially be controlled by the systemic chemotherapy, as would more distant sites, we did not electively irradiate uninvolved regional nodes. Supporting this assumption we observed no isolated regional recurrences, which is consistent with findings of other studies which excluded elective nodal irradiation [33,34]. In a phase I radiation dose escalation study of 34 unresectable or incompletely resected PC patients treated with RT plus concurrent gemcitabine, McGinn et al. [33] reduced RT fields (PTV = GTV + 1 cm) which was even smaller than our current definition, and observed only 3 (8.8%) regional failures. The approach excluding the elective irradiation of regional nodes is further supported by the current radiosurgical practice in PC [34], which permits only milimetric margins around the GTV with loco-regional control and survival rates similar or even better to that of conventional larger field external beam RT studies. Taken together, we advocate the idea not to irradiate elective nodal irradiation at least to an attempt to decrease C-CRT-related toxicity and possibly to escalate the RT dose to more effective levels with highly conformal irradiation techniques until this issue is enlightened with randomized trials.

## Conclusions

We have investigated the potential prognostic value of the GTV delineated by co-registered contrast-enhanced CT/FDG-PET-CT-based RTP on outcomes in patients with LAPC. The superior OS, LRPFS and PFS observed in GTV_L _patients over GTV_G _ones suggests a potential for FDG-PET-CT-defined GTV size in predicting outcomes of LAPC patients treated with definitive C-CRT. Since it is neither easy nor appropriate to conclude firmly with findings from such a small study population, results presented here should better be interpreted with caution and warrants to be addressed in future trials with larger cohorts.

## Competing interests

We have no personal or financial competing interest and have not entered into any agreement that could interfere with our access to the research data, or upon our ability to analyze the data independently, to prepare manuscripts, or to publish them.

## Authors' contributions

Study conception and design: CP, ET. Provision of study materials or patients: CP, ET, MR. Collection and assembly of data: CP, ET, CO. Data analysis and interpretation: CP, ET, MR, US. Manuscript writing: CP, ET, US. Final approval of manuscript: CP, ET, CO, MR, US. All authors read and approved the final manuscript.
